# Perioperative Changes in Plasma Nitrite and IL-6 Levels Predict Postoperative Atrial Fibrillation (POAF) and Acute Kidney Injury (AKI) after Cardiac Surgery

**DOI:** 10.3390/antiox13080971

**Published:** 2024-08-09

**Authors:** Matthew A. Fischer, Kimberly Howard-Quijano, Nobel Chenggong Zong, Ji Youn Youn, Norika Mengchia Liu, Jennifer Scovotti, Tristan Grogan, Aman Mahajan, Hua Cai

**Affiliations:** 1 Department of Anesthesiology & Perioperative Medicine, David Geffen School of Medicine, University of California Los Angeles (UCLA), Los Angeles, CA 90095, USA; mfischer@mednet.ucla.edu (M.A.F.);; 2 Department of Anesthesiology & Perioperative Medicine, University of Pittsburgh, Pittsburgh, PA 15260, USA; howardquijanokj@upmc.edu

**Keywords:** ROS, nitrite, nitric oxide (NO), post-operative complications, atrial fibrillation, acute kidney injury

## Abstract

**Background:** Postoperative atrial fibrillation (POAF) and acute kidney injury (AKI) are common yet significant complications after cardiac surgery, with incidences of up to 40% for each. Here, we assessed plasma nitrite and serum interleukin-6 (IL-6) levels before and after cardiac surgery to quantify the extent to which oxidative stress and inflammation contribute to POAF and AKI occurrence. **Methods:** We prospectively enrolled 206 cardiac surgical patients. Plasma nitrite and serum IL-6 levels were determined preoperatively and at 24 h, 48 h and 72 h postoperatively. The patients had continuous EKG monitoring for occurrence of POAF, while daily serum creatinine was measured for determination of stage 1 + AKI. **Results:** Postoperatively, 78 (38%) patients experienced AF, and 47 (23%) patients experienced stage 1 + AKI. *POAF analysis*: Age, ACE-inhibitor use, valve surgery and percent change in baseline plasma nitrite at 24 h postoperatively were associated with POAF in multiple logistic regression analysis. The inclusion of this new biomarker significantly improved the POAF prediction model (AUC 0.77 for clinical risk factors alone, to AUC 0.81). *AKI analysis*: A history of diabetes mellitus was associated with AKI in multiple logistic regression analysis, and the addition of preoperative IL-6 levels improved the prediction model for AKI occurrence (AUC 0.69 to AUC 0.74). **Conclusions:** We previously observed selective upregulation of NADPH oxidase isoform 4 (NOX4) in patients with AF, a critical causal role of NOX4 for AF in zebrafish and a robust inhibitory effect of nitric oxide (NO) on NOX4. Our data innovatively demonstrate that a reduction in circulating nitrite levels, likely implicative of elevated NOX4-mediated oxidative stress, independently associates with POAF and improves POAF prediction, whereas the inclusion of circulating IL-6 levels improves the prediction model for AKI. Therefore, therapeutic strategies to mitigate these pathophysiological sequalae of surgical stress may reduce the incidence of severe postoperative complications of POAF and AKI.

## 1. Introduction

Cardiac surgery is known to trigger the development of severe postoperative complications [1,2], including postoperative atrial fibrillation (POAF) and acute kidney injury (AKI), which significantly worsen patient outcomes and increase the cost of healthcare [3,4]. POAF is typically transient, with a peak incidence between postoperative days two and four, and complicates up to 40% of cardiac surgeries [3]. POAF after cardiac surgery is associated with increased short- and long-term morbidity and mortality as well as an eightfold increase in the risk of subsequent atrial fibrillation (AF) [3]. The occurrence of POAF is understood to be secondary to the physiologic stress of surgery superimposed on pre-existing atrial substrate [3]. Clinical risk factors include age, male sex, history of paroxysmal atrial fibrillation, obesity, hypertension and congestive heart failure [3].

Acute kidney injury is another significant complication after cardiac surgery and varies from 5% to 42% in incidence [4]. A large study of over 50,000 patients undergoing major inpatient surgery reported an incidence of 39% using the RIFLE (Risk, Injury, Failure, Loss of kidney function, and End-stage kidney disease) criteria [5]. Cardiac surgery-associated acute kidney injury (CSA-AKI) is the second most common cause of AKI after sepsis in critical care units and is independently associated with increased morbidity and mortality [4]. The severity of AKI can be graded by a variety of criteria: AKIN (Acute Kidney Injury Network), RIFLE and KDIGO (Kidney Disease: Improving Global Outcomes) [6]. KDIGO has a greater sensitivity to detect AKI and is the current recommendation by consensus guideline [7]. Clinical risk factors for CSA-AKI include age, female sex, diabetes mellitus, pre-existing chronic kidney disease and congestive heart failure. Similar to POAF, the known clinical risk factors associated with CSA-AKI do not suggest any known causal mechanisms, though perioperative hemodynamic perturbations, nephrotoxins and physiologic stress are contributory to increased incidence of AKI [4,6]. Improved mechanistic understanding of the pathophysiology of POAF and AKI could yield opportunities for risk mitigation.

Since physiologic stress/oxidative stress has a significant impact on both POAF and AKI, we analyzed various biomarkers that capture the consequences of oxidative stress induced by cardiac surgery-triggered ischemic insults, including changes in circulating nitrite levels as a readout of NO and circulating IL-6 levels as a readout for inflammatory activation. Serum interleukin-6 (IL-6) is a pro-inflammatory cytokine whose magnitude is associated with the extent of tissue injury and degree of cardiogenic shock [8]. Though many inflammatory cytokines are elevated after surgery, we chose to measure IL-6 because it has been associated with the degree of surgical trauma [9]. Plasma nitric oxide (NO) inhibits NADPH oxidase 4 (NOX4), a major source of cardiac oxidative stress [10,11]. We hypothesized that reduced circulating nitrite levels likely reflect the extent of NOX4-mediated oxidative stress [10,11] associated with surgery and cardiopulmonary bypass. Of note, we have previously shown that NOX4 is selectively upregulated in patients with AF [12] and that NOX4-derived oxidative stress triggers an AF-like cardiac arrhythmia in a zebrafish model [13]. To assess the association of circulating NO levels with the occurrence of POAF, we measured nitrite levels in plasma as a readout of the tissue bioavailability of NO [14]. Together, IL-6 and nitrite reflect the inflammatory response and oxidative stress in this particular disease setting, respectively, associated with the physiologic response to cardiac surgery and cardiopulmonary bypass.

In this study, we seek to understand the value of specific biomarkers of inflammation and oxidative stress in predicting the occurrence of POAF and AKI after cardiac surgery. We analyzed POAF as the primary outcome of interest in this study, with AKI studied as a secondary outcome to further support the impact of inflammation and oxidative stress on another significant adverse postoperative complication. We found a statistically significant improvement in POAF and AKI prediction with the inclusion of circulating nitrite and IL-6 levels, respectively, in prediction models. These findings, for the first time, innovatively quantify the contribution of oxidative stress and inflammation underlying the physiologic stress of surgery to amplify postoperative complications and demonstrate the significant predictive impacts of these postoperative physiologic derangements—on top of known preoperative clinical risk factors—enabling more powerful detection and management of these severe postoperative complications.

## 2. Materials and Methods

### 2.1. Patient Selection

After institutional review board approval and written informed consent, adult patients scheduled for cardiac and aortic surgery on cardiopulmonary bypass were prospectively enrolled at Ronald Reagan Medical Center at the University of California, Los Angeles. Surgical procedures included aortic valve replacement, mitral valve repair/replacement, coronary artery bypass grafting and combined coronary artery bypass/valve surgery. Patients meeting the inclusion criteria were enrolled consecutively. We chose to enroll patients undergoing a variety of surgical procedures because this reflects the patient population we care for in the university setting, and for which we would like to better understand the impact of inflammation and oxidative stress on postoperative complications. Patients with chronic AF and congenital heart disease (except bicuspid aortic valve) were excluded. Established clinical risk factors for POAF [3,15] and AKI [4,16] were obtained from the surgical preoperative history and physical, operative note and perfusion summary. These data are presented in Table 1 and Table 2 for POAF and AKI, respectively. Patients on preoperative dialysis were excluded from the AKI cohort.

### 2.2. Occurrence of Postoperative Atrial Fibrillation and Acute Kidney Injury

Patients were followed prospectively for the occurrence of POAF. POAF was defined as any clinician diagnosis of AF at least 30 s in duration from postoperative ICU admission until hospital discharge. We defined AF as an occurrence [15] because we would like to study factors contributing to the development of AF rather than factors associated with sustaining AF for longer duration of time. Patients in the cardiac surgical intensive care unit had continuous electrocardiogram monitoring for electronic and clinical diagnosis of AF via physician readout. In addition, patients transferred out of the ICU had continuous EKG monitoring via telemetry until discharge from the hospital. EKG data was reviewed manually for occurrences of POAF in a double-blinded fashion with respect to the biomarker data.

AKI was determined as any patient demonstrating stage 1 or worse AKI by the KDIGO criteria [7] using only serum creatinine values: a serum creatinine increase ≥0.3 mg/dL in the first 48 h or ≥1.5x increase in serum creatinine from baseline in the first 7 days. Serum creatinine values were measured daily for all patients until discharge. Reliable hourly urine output data for these patients was not available, as AKI was our secondary outcome. Serum creatinine data was obtained retrospectively from the electronic medical record using the UCLA perioperative data warehouse [17].

### 2.3. Plasma Biomarker Data

Whole blood samples were collected in EDTA tubes preoperatively and at 24, 48 and 72 h postoperatively. Plasma samples were freshly prepared and subjected to determine nitrite levels using Griess reagent (see “Section 2.4” below). Serum IL-6 and BNP levels were measured by the clinical lab at the UCLA Medical Center using routine clinical laboratory testing.

### 2.4. Determination of Plasma Nitrite Levels

The plasma from patient blood samples was separated by centrifugation at 2000× *g* for 20 min prior to being treated with protamine sulfate salt (#P4020, MilliporeSigma, Burlington, MA, USA) for 5 min at room temperature to neutralize the heparin. The deheparinized plasma samples were subjected to nitrite assay using a Griess reagent kit (#30100, Biotium, Fremont, CA, USA). In brief, 150 µL of deheparinized plasma or sodium nitrite as a standard (at concentrations of 1.25, 2.5, 5 and 10 µM) were mixed with 150 µL of Griess reagent working solution. After incubation at room temperature for 5 min, optical density at 540 nm was determined using a microplate reader (CLARIOstar, BMG Labtech, Ortenberg, Germany). Nitrite levels were calculated by the standard curve generated using sodium nitrite. Circulating levels of nitrite are considered an indicator of tissue NO bioavailability.

### 2.5. Statistical Analysis

Patient characteristics and biomarkers were summarized between patients who had POAF and/or AKI versus those who did not. Continuous data were summarized by mean and standard deviation (SD), while categorical data were summarized by count and frequency unless otherwise noted. The statistical significance of these data was formally compared using a chi-square test or *t*-test. For assessing the biomarkers at each time point between POAF/no-POAF or AKI/no-AKI, due to distributional assumptions, we summarized them over time using medians and quartiles and compared them using the Mann–Whitney U test. Plasma nitrite levels and serum IL-6 levels demonstrated skewed distributions with the Kolmogorov–Smirnov normality test, *p* < 0.05. Percent change in plasma nitrite levels from baseline to 24 h post-surgery was normally distributed with the Kolmogorov–Smirnov normality test, *p* > 0.05. For our primary aim, given a sample size of 78 patients who experienced POAF and 128 who did not (POAF incidence 38%), we are adequately powered to detect biomarker effect sizes of at least 0.41 between groups. This is assuming a two-sample t-test with a two-tailed alpha = 0.05.

Multiple logistic regression of POAF and AKI occurrence was performed on all predictors, with *p* < 0.05, in univariate analysis. For AKI occurrence, IL-6 at 72 h was omitted from multiple regression analysis due to the relatively fewer patients who had this measurement, and the finding that patients experiencing AKI were more likely to have an arterial line or central venous line for clinical management and thus were more likely to have the samples drawn.

POAF and AKI prediction models were constructed to demonstrate the relative addition of our biomarkers to event occurrence rather than clinical risk prediction. Features with *p*-value < 0.25 in univariate analysis were considered as candidates for each model [18]. Stepwise logistic regression was then performed to select features in JMP version 16 (Cary, NC, USA). Features for the POAF model were selected using the minimum Bayesian information criterion. Features for the AKI model were selected using the minimum Akaike information criterion because the minimum Bayesian information criterion selected only one feature. The models were then constructed using fivefold cross-validation repeated 10 times using the caret package in the R program [19]. Model confidence intervals were obtained using the pROC package in R [20], and the statistical significance of differences between prediction models was calculated using DeLong’s test. Box plots and ROC curve figures were generated with the ggplot2 package in R [21]. All statistical analysis unless otherwise stated was performed using R version 4.1.1 (Vienna, Austria), and *p* < 0.05 was considered statistically significant.

## 3. Results

We enrolled 206 patients in this study from November 2013 to May 2018, and levels of IL-6, nitrite and BNP were determined from blood samples from all the patients. The incidence of POAF and stage 1 + AKI were 38% and 23%, respectively. Table 1 and Table 2 demonstrate the distribution of clinical risk factors between POAF and stage 1 + AKI groups, respectively. There were 99 patients who experienced either POAF or stage 1 + AKI, and 26 patients who experienced both POAF and stage 1 + AKI.

### 3.1. Univariate Analysis of Clinical Risk Factors for Postoperative Atrial Fibrillation

First, we assessed the effect of known clinical risk factors in our cohort before determining the enhanced predictive effect of our biomarkers on the risk of POAF. We assessed the statistical significance of the following established clinical risk factors [3,15] for POAF in our cohort: age, sex, body mass index (BMI), history of paroxysmal AF, hypertension, diabetes mellitus, congestive heart failure, preoperative angiotensin-converting enzyme-inhibitor (ACEI) use, preoperative angiotensin receptor blocker (ARB) use, preoperative beta blocker use and valve surgery. We also analyzed preoperative statin use due to its anti-inflammatory effects [22], NO regulating effects [23] and association with decreased POAF risk in some studies [24]. Additionally, we analyzed cardiopulmonary bypass time and combined surgery (two or more of the following surgeries: valve surgery, coronary artery bypass grafting, aortic surgery and myomectomy) to examine the effects of surgical complexity on POAF risk. The results of this univariate analysis are shown in Table 1. Age, ACEI use, ARB use, and valve surgery were statistically significant in univariate analysis.

### 3.2. Univariate Analysis of Clinical Risk Factors for Acute Kidney Injury

Similarly, we assessed the effect of established clinical risk factors for acute kidney injury before determining the enhancing effect of our biomarkers on predicting stage 1 or greater AKI. The established clinical risk factors [4,16] examined include age, sex, preoperative serum creatinine, BMI, hypertension, diabetes mellitus, congestive heart failure, preoperative ACEI use and preoperative ARB use. We also analyzed the effect of preoperative beta blocker and statin use on AKI. In addition, we examined the effect of surgical complexity and type of surgery on the risk of AKI by including valve surgery, combined surgeries and cardiopulmonary bypass time in the analysis. The results of this univariate analysis are shown in Table 2. Age, preoperative creatinine, diabetes mellitus, preoperative beta blocker use, and preoperative statin use were statistically significant in univariate analysis.

### 3.3. Univariate Analysis of Serum and Plasma Biomarkers for POAF

**Figure 1:**
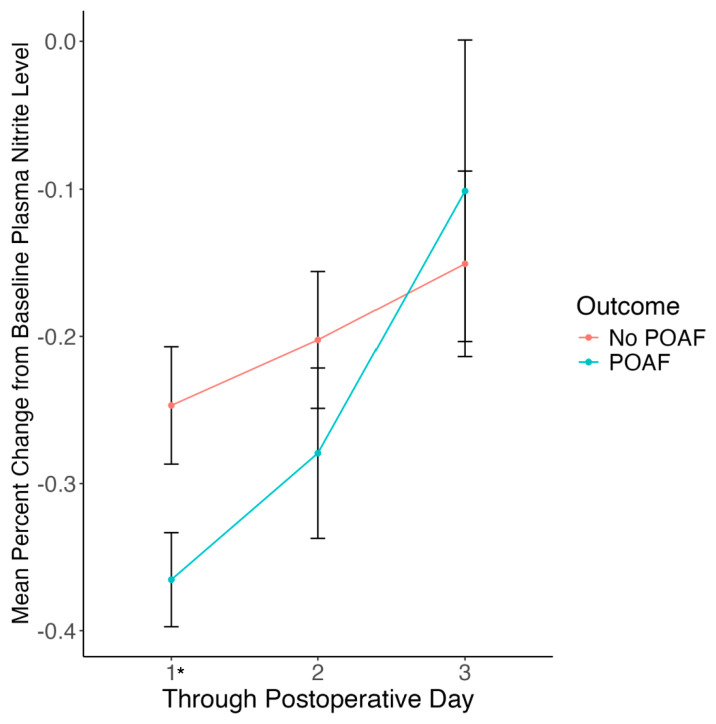
Percent Change from Baseline Plasma Nitrite Level by POAF Occurrence. These line plots demonstrate the mean percent change in baseline plasma nitrite level through postoperative days 1, 2 and 3 for patients who experience POAF and those who do not. The error bars display the standard error of the mean. The mean percentage change in plasma nitrite level from baseline is statistically significant through postoperative day 1 (*p*-value = 0.033) but not through postoperative days 2 or 3. Statistically significant results are indicated by *.

**Figure 2:**
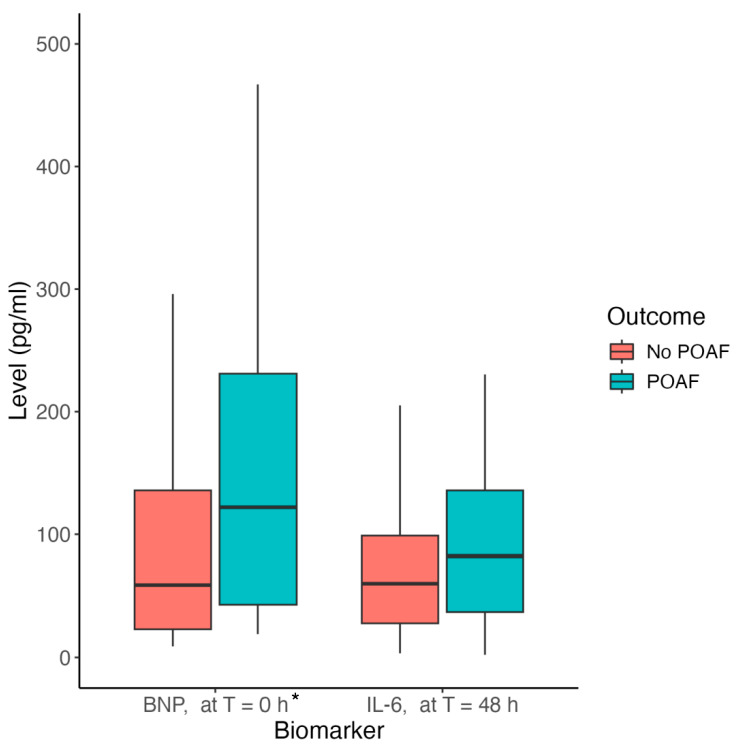
Biomarker Level by POAF Occurrence. These box plots demonstrate the association of preoperative BNP levels (*p*-value = 0.001) and postoperative day 2 IL-6 levels (*p*-value = 0.126) with POAF occurrence. Statistically significant results indicated by *.

The distributions of biomarkers for patients who did or did not experience POAF are shown in Table 3 along with the number of samples analyzed for each group. We analyzed the association of circulating nitrite and IL-6 levels preoperatively as well as at 24, 48 and 72 h postoperatively with POAF. In addition, we analyzed preoperative BNP and percent change in nitrite in the first 24 h. Percent change in baseline plasma nitrite at 24 h and preoperative BNP were statistically significant in univariate analysis. Figure 1 shows a line plot comparing the mean percent change in baseline plasma nitrite by postoperative day for patients who experienced POAF compared to those who did not. This figure demonstrates that patients who had a greater percent decrease in plasma nitrite levels in the first 24 h had an increased risk of POAF. Figure 2 compares preoperative serum BNP and serum IL-6 levels at 48 h between patients who experienced POAF and those who did not. BNP was statistically significant (*p*-value = 0.033) between patients experiencing POAF and those who did not. Although IL-6 at 48 h was not statistically associated with POAF (*p*-value = 0.126), it did improve POAF prediction, as detailed in the “POAF Prediction Model” section.

### 3.4. Multiple Regression Analysis of Serum and Plasma Biomarkers for POAF

We then performed multiple logistic regression of POAF using clinical and biomarker predictors that had *p* < 0.05 in univariate analysis. Multiple logistic regression of POAF occurrence demonstrated that age, ACEI use, valve surgery and percent change of plasma nitrite levels at 24 h were statistically significant after accounting for other risk factors. The results of this multiple regression analysis are shown in Table 4.

### 3.5. POAF Prediction Model

**Figure 3:**
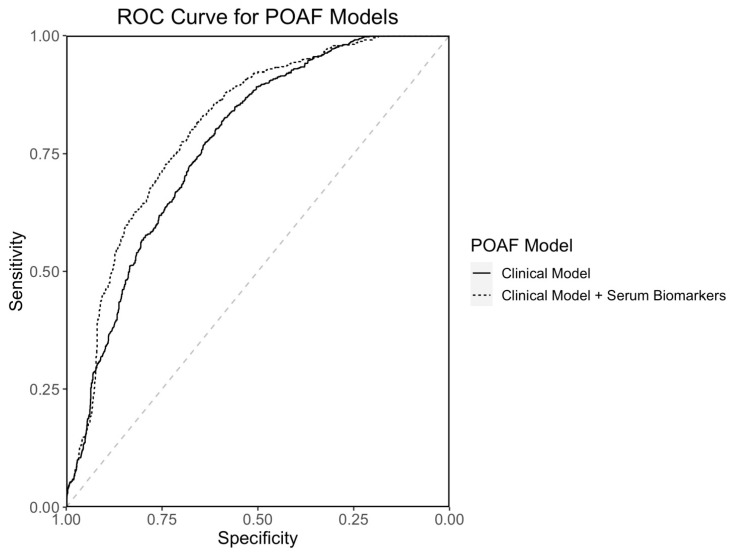
ROC Curve for POAF Prediction Model. ROC curves are shown for the POAF prediction model in Table 5 (age, ACE inhibitor use, valve surgery, IL-6 level at 48 h and percent change from baseline plasma nitrite levels in the first 24 h) and a POAF prediction model with clinical predictors only (age, ACE inhibitor use and valve surgery). The AUC for the clinical model is 0.77 (95% CI 0.75–0.79) and the AUC for the clinical model with IL-6 at 48 h + %Δ Nitrite at 24 h is 0.81 (95% CI 0.79–0.83). The improvement in AUC for the clinical model with biomarkers is statistically significant by DeLong’s test with *p*-value = 0.0093.

The POAF prediction model is shown in Table 5. The model contains age, ACEI use, valve surgery, serum IL-6 levels at 48 h and percent change of plasma nitrite levels at 24 h. The AUC of the POAF prediction model is 0.81 (95% CI 0.79–0.83) and the ROC curve is shown in Figure 3. The AUC of the POAF prediction model without biomarkers (predictors: age, ACEI use and valve surgery) was 0.77 (95% CI 0.75–0.79). The 0.81 AUC of the POAF prediction model shows good discrimination and an improvement over the model with only clinical risk factors. The difference between the POAF model with biomarkers and the model without was significant by DeLong’s test with *p*-value = 0.0093, demonstrating the statistical significance of the addition of serum IL-6 levels at 48 h and percent change of plasma nitrite levels at 24 h to the model of POAF occurrence.

### 3.6. Univariate Analysis of Serum and Plasma Biomarkers for Stage 1 + AKI

The distribution of biomarkers for patients who did or did not experience Stage 1 + AKI is shown in Table 6, along with the number of samples analyzed for each group. We analyzed the association of circulating nitrite and IL-6 levels preoperatively as well as at 24, 48 and 72 h postoperatively with AKI. In addition, we analyzed preoperative BNP and percent changes in plasma nitrite levels in the first 24 h. Preoperative serum IL-6 levels, serum IL-6 levels at 72 h postoperatively and preoperative BNP levels were significant in univariate analysis.

### 3.7. Multiple Regression Analysis of Serum and Plasma Biomarkers for Stage 1 + AKI

We next performed multiple logistic regression of stage 1 + AKI using clinical and biomarker predictors that had *p* < 0.05 in univariate analysis. Multiple logistic regression of stage 1 + AKI occurrence demonstrated that a history of diabetes mellitus was statistically significant after accounting for other risk factors. The results of this multiple regression analysis are shown in Table 7.

### 3.8. Stage 1 + AKI Prediction Model

**Figure 4:**
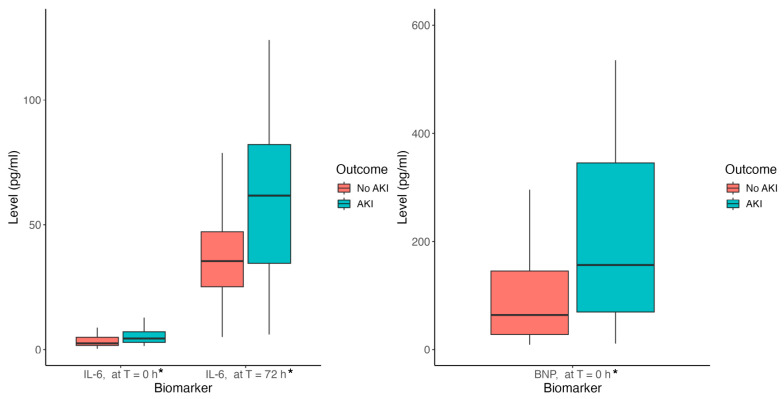
Biomarker Level by AKI Occurrence. These box plots demonstrate the association of preoperative IL-6 levels (*p*-value < 0.001), postoperative day 3 IL-6 levels (*p*-value = 0.002) and preoperative BNP levels (*p*-value < 0.001) with stage 1 or greater AKI occurrence. Statistically significant results are indicated by *.

**Figure 5:**
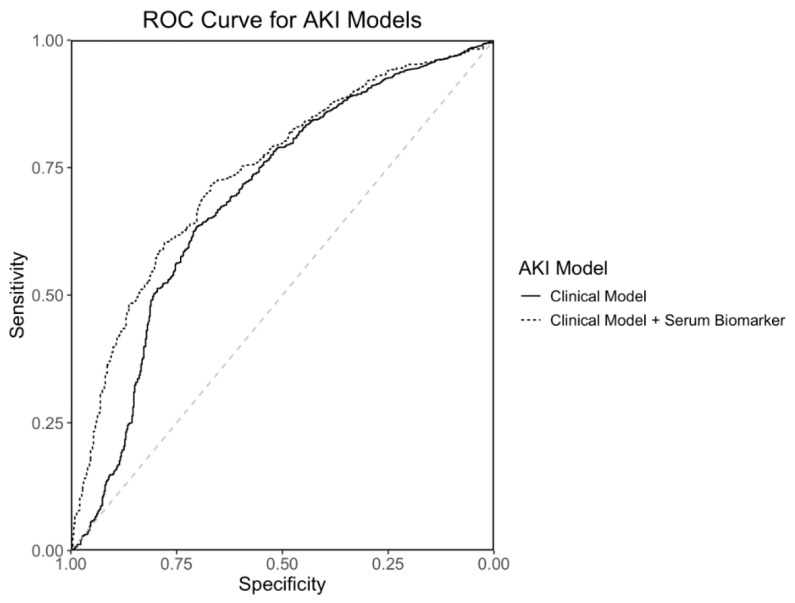
ROC Curve for AKI Prediction Model. ROC curves are shown for the AKI prediction model in Table 8 (diabetes mellitus, preoperative IL-6 level and cardiopulmonary bypass time) and an AKI prediction model with clinical predictors only (diabetes mellitus and cardiopulmonary bypass time). The AUC for the clinical model is 0.69 (95% CI 0.66–0.72) and the AUC for the clinical model with IL-6 is 0.74 (95% CI 0.71–0.77). The improvement in AUC for the clinical model with biomarkers is statistically significant by DeLong’s test with *p*-value = 0.016.

The stage 1 + AKI prediction model is shown in Table 8. The AKI prediction model comprised diabetes mellitus, preoperative serum IL-6 levels and cardiopulmonary bypass time. The preoperative serum IL-6 levels, serum IL-6 levels at 72 h postoperatively and preoperative serum BNP levels are shown by stage 1 + AKI occurrence in Figure 4. The AUC of the AKI prediction model in Table 8 is 0.74 (95% CI 0.71–0.77), and the ROC curve is shown in Figure 5. The AUC of the AKI prediction model without biomarkers (predictors: diabetes mellitus and cardiopulmonary bypass time) is 0.69 (95% CI 0.66–0.72). The difference between the AKI model with the IL-6 biomarker and the model without is significant by DeLong’s test with *p*-value = 0.016, demonstrating the statistical significance of the addition of preoperative serum IL-6 levels to the model of AKI occurrence. 

## 4. Discussion

Atrial fibrillation and acute kidney injury are complex disease processes with multiple patient-specific and environmental factors related to their occurrence [3,6]. In this study, we demonstrate the association of circulating nitrite and IL-6 levels with two significant postoperative complications after cardiac surgery that are highly prevalent, occurring in up to 40% of all cardiac surgical patients [25]. These biomarkers reflect a measurable component of the preoperative and postoperative oxidative stress and inflammation in patients undergoing cardiac surgery on cardiopulmonary bypass. Our main findings are: (1) a greater percent decrease in plasma nitrite levels in the first 24 h was independently associated with the occurrence of POAF; (2) the AUC for the POAF model was significantly improved from 0.77 to 0.81 with the inclusion of reduced circulating nitrite levels as a reflection of increased oxidative stress; (3) the AUC of the AKI prediction model was improved from 0.69 to 0.74 with the inclusion of increased circulating IL-6 levels.

Multiple regression analysis (Table 4) demonstrated that the percent change in plasma nitrite levels, age, preoperative ACEI use, and valve surgery were independently associated with POAF. Age, preoperative ACEI use, and valve surgery have been shown to be associated with POAF in prior studies [3,15] and we observe consistent results in this cohort. Decreased circulating levels of nitrite indicate reduced bioavailability of NO, which is known to inhibit NOX4 [10,11], a significant source of cardiac oxidative stress, to trigger an AF-like arrhythmic phenotype in zebrafish and is known to be upregulated in patients with AF [12,13]. In other words, decreased plasma nitrite levels are consistent with decreased NOX4 inhibition by NO, which likely increases the risk of AF [12,26]. As mentioned, we have previously demonstrated that upregulated NOX4 expression is present in heart transplantation patients with AF [12] and that RNA-based induction of NOX4 induces an AF-like arrhythmic phenotype in zebrafish [13]. This critical finding suggests that increased oxidative stress immediately after cardiac surgery increases the risk of POAF. Figure 1 shows the percent change from baseline plasma nitrite levels by POAF occurrence for postoperative days 1 through 3. The timing of oxidative stress precedes the occurrence of POAF, which typically occurs between postoperative days two and four [3]. Of note, the inhibitory effects of NO on NOX4 are derived from endothelial production of NO from eNOS, leading to protective effects on cardiomyocytes [11].

The POAF prediction model was created to demonstrate the relative contribution and efficacy of the POAF predictors (Table 5) in explaining event occurrence. Due to the biomarker measurements occurring after surgery, their utility in clinical risk prediction is less relevant than the demonstration of their contribution to POAF occurrence. For this reason, we compared biomarkers to established clinical risk factors to show their relative weight and necessity within models explaining event occurrence, rather than testing their inclusion in established POAF risk scores to improve preoperative prediction. In addition, the finding that the AUC for the POAF model with biomarkers is statistically significantly higher than the POAF model without biomarkers demonstrates that the inclusion of circulating nitrite and IL-6 biomarkers improves the identification of event occurrence and is important to the model. The ROC curves for the POAF model with and without biomarkers are shown in Figure 3. This finding supports the conclusion that the percent change in plasma nitrite levels in the first 24 h is significant for predicting POAF occurrence. It has been previously shown that ROS increases after myocardial reperfusion [27], and this increased ROS is hypothesized [3] to play a role in mediating ischemia/reperfusion sensitization of the atria to POAF. Here, we show that a greater percent decrease in plasma nitrite in the first 24 h is associated with POAF occurrence. The association of POAF with increased postoperative levels of IL-6 is consistent with other studies [28,29] in cardiac surgical patients.

Multiple regression analysis of AKI demonstrated that a history of diabetes mellitus is independently associated with stage 1 + AKI. Our analysis is consistent with other studies showing that diabetes mellitus is a significant independent risk factor for AKI after cardiac surgery. Preoperative serum IL-6 reached near statistical significance (*p*-value = 0.069) with stage 1 + AKI in multiple logistic regression and improved the prediction of stage 1 + AKI occurrence in the prediction model. Interestingly, preoperative plasma IL-6 has been shown to be associated with AKI after cardiac surgery in pediatric patients but not in adult patients [30].

The stage 1 + AKI prediction model was similarly created to assess the relative contribution and efficacy of the AKI predictors (Table 8) in accounting for AKI occurrence. In this analysis, we compared the measured biomarkers to established clinical risk factors for AKI to show their weight and utility within models explaining event occurrence, rather than testing their added value to established AKI risk scores to improve preoperative prediction. For the AKI model, the biomarker of interest is preoperative circulating IL-6 levels. Though the primary goal of constructing this model was to quantify the contribution of IL-6 to event occurrence, this model could theoretically be used before surgery to assess the risk of postoperative AKI. What is unknown, however, is if IL-6 levels taken at the time of routine preoperative labs, which typically occur a few weeks before elective cardiac surgery, would be consistent with IL-6 levels measured on the day of surgery and would be similarly predictive of AKI. Further research on this subject is needed to further elucidate the role of preoperative circulating IL-6 levels in predicting postoperative AKI. The finding that the AUC for the stage 1 + AKI model with biomarkers is statistically significantly higher by DeLong’s test compared to the AKI model without biomarkers demonstrates that the inclusion of preoperative IL-6 improves the identification of event occurrence and is important to the model. The ROC curves for the stage 1 + AKI model with and without biomarkers are shown in Figure 5. This suggests that baseline IL-6 is important for the prediction of stage 1 + AKI occurrence.

This research demonstrates the relative contribution of inflammation and oxidative stress to the occurrence of POAF and AKI after cardiac surgery. The significance of this research is that it helps quantify the physiological stress of patients undergoing cardiac surgery and cardiopulmonary bypass that leads to these adverse perioperative outcomes. The improved prediction suggests potential modulation of the stress response to surgery may improve the occurrence of these adverse outcomes. Further research on this topic is required. In addition, preoperative inflammation was found to improve the prediction of AKI. This preoperative information may help risk-stratify patients undergoing cardiac surgery. For example, patients with an elevated baseline inflammatory state may benefit from transcatheter aortic valve replacement (TAVR) over open surgical repair, due to the lower incidence of AKI associated with TAVR [31]. In addition, ACEI use before cardiac surgery was associated with a decreased risk of POAF (OR 0.31, 95% CI 0.14–0.72) and is a potential strategy for risk mitigation, though further research is needed given this is an observational cohort study.

Limitations of this study include the possibility that biomarker data were not missing at random. In other words, there may be factors related to POAF or AKI occurrence that also affect whether biomarker samples were obtained. In our study, we observed that patients who experienced AKI were more likely to have biomarker samples available at 72 h because they still had invasive vascular access due to their adverse outcome. In addition, the 72 h time point is after the occurrence of many POAF and AKI events and so was excluded from multiple regression analysis and prediction models. Preoperative samples and 24 h postoperative samples were obtained from all the patients, and any exclusions were secondary to the samples being hemolyzed. Compared to the 72 h time point, samples obtained 48 h postoperatively had relatively few missing samples, with the majority of these being secondary to hemolysis. In addition, we did not perform a time-to-event analysis, instead opting for a simpler analysis by postoperative day due to our primary goal of demonstrating the association and relative contribution of these biomarkers in explaining event occurrence. However, given that the statistically significant biomarkers and those used in the prediction models were preoperative or from within 48 h postoperatively, the biomarker samples would be before or on the same day of the typical peak incidence of POAF and AKI.

In conclusion, we demonstrated that biomarkers for the extent of inflammation and oxidative stress are independently associated with POAF. We present the critical findings that a greater decrease in circulating plasma nitrite levels in the first 24 h after surgery is independently associated with POAF. This significant observation suggests that oxidative stress likely precedes POAF occurrence, and also demonstrates that a surgically induced risk factor, together with known preoperative clinical risk factors, work synergistically to drive AF occurrence in the postoperative period. Therapeutic interventions to mitigate the extent of postoperative oxidative stress may therefore reduce the incidence of POAF after cardiac surgery. In addition, preoperative IL-6 levels improve the prediction of stage 1 + AKI. These findings quantify how the physiological stress of surgery is associated with each adverse outcome. Further research is needed to understand how similar patients can produce variable stress responses to surgery, which may suggest a genetic or genomic predisposition producing this variable stress response. Improved prediction and understanding of which patients will respond to surgery with increased oxidative stress and an elevated inflammatory response would identify patients at increased risk for adverse outcomes and reveal mechanisms and preventive strategies to attenuate the development of POAF and AKI.

## Figures and Tables

**Table 1:** Characteristics of Patient Population by POAF. The number and prevalence of clinical risk factors are displayed in this table for the patients who experienced postoperative atrial fibrillation and for those who did not.

Characteristic	No POAF	POAF	*p*-Value
Number of Patients	128 (62%)	78 (38%)	----
Male	96 (75%)	59 (76%)	1.000
Age, mean ± SD	59 ± 14	70 ± 10	**<0.0001**
BMI, mean ± SD	27 ± 5	28 ± 6	0.691
History Paroxysmal Atrial Fibrillation	2 (2%)	2 (3%)	1.000
Hypertension	81 (63%)	59 (76%)	0.091
Diabetes Mellitus	29 (23%)	21 (27%)	0.599
Congestive Heart Failure	26 (20%)	19 (24%)	0.612
ACE Inhibitor	44 (34%)	15 (19%)	**0.030**
Angiotensin Receptor Blocker	20 (16%)	23 (29%)	**0.028**
Beta Blocker	66 (52%)	39 (50%)	0.941
Statin	72 (56%)	55 (71%)	0.058
Valve Surgery	76 (59%)	58 (74%)	**0.042**
Combined Surgery	34 (27%)	30 (38%)	0.102
Cardiopulmonary Bypass Time, mean ± SD	145 ± 70	144 ± 54	0.862

**Table 2:** Characteristics of Patient Population by AKI. The number and prevalence of clinical risk factors are displayed in this table for the patients who experienced stage 1 or greater AKI and for those who did not.

Characteristic	No AKI	Stage 1 + AKI	*p*-Value
Number of Patients	159 (77%)	47 (23%)	----
Male	115 (72%)	40 (85%)	0.112
Age, mean ± SD	62 ± 14	66 ± 12	**0.039**
Preoperative Creatinine, mean ± SD	1.1 ± 0.8	1.5 ± 1.3	**0.035**
BMI, mean ± SD	27 ± 5	28 ± 6	0.277
Hypertension	106 (67%)	34 (72%)	0.579
Diabetes Mellitus	29 (18%)	21 (45%)	**<0.001**
Congestive Heart Failure	37 (23%)	8 (17%)	0.478
ACE Inhibitor	48 (30%)	11 (23%)	0.471
Angiotensin Receptor Blocker	32 (20%)	11 (23%)	0.778
Beta Blocker	74 (47%)	31 (66%)	**0.030**
Statin	90 (57%)	37 (79%)	**0.010**
Valve Surgery	109 (69%)	25 (53%)	0.077
Combined Surgery	46 (29%)	18 (38%)	0.299
Cardiopulmonary Bypass Time, mean ± SD	141 ± 65	155 ± 60	0.160

**Table 3:** Biomarkers by POAF Occurrence. Median values are reported for plasma nitrite, percent change in plasma nitrite, IL-6, and BNP levels by POAF occurrence for each listed time point. *p*-values have been calculated using the Mann–Whitney U test for all biomarkers except percent change in plasma nitrite. Percent change in plasma nitrite from baseline to postoperative day 1 was normally distributed (Kolmogorov–Smirnov normality test, *p* > 0.05), thus, the *p*-value was calculated using Student’s *t*-test assuming unequal variance.

	No POAF				POAF				
Serum Biomarker	Sample Size	25th Percentile	Median	75th Percentile	Sample Size	25th Percentile	Median	75th Percentile	*p*-Value
Nitrite, at T = 0 h	122	3.1	4.5	8.5	77	3.2	4.9	10.2	0.431
Nitrite, at T = 24 h	126	1.9	2.9	5.2	78	1.9	3.1	5.6	0.891
Nitrite, at T = 48 h	115	2.0	2.9	5.7	67	2.0	3.0	5.7	0.734
Nitrite, at T = 72 h	66	1.9	2.4	4.2	40	2.2	2.9	5.8	0.131
% Δ Nitrite, T = 0 to T = 24 h	122	−58.6%	−32.3%	1.9%	77	−58.6%	−40.1%	−21.2%	**0.033**
IL-6, at T = 0 h	123	1.6	2.6	5.0	76	2.0	3.1	5.0	0.143
IL-6, at T = 24 h	122	31.4	62.1	119.4	76	42.0	82.9	148.8	0.089
IL-6, at T = 48 h	113	27.8	60.2	98.9	66	37.0	82.4	135.8	0.126
IL-6, at T = 72 h	68	26.2	37.4	53.5	40	26.2	44.0	72.7	0.423
BNP, at T = 0 h	124	23.0	59.0	135.8	77	43.0	122.0	231.0	**0.001**

**Table 4:** Multiple Logistic Regression of Clinical and Biomarker POAF Predictors. Clinical characteristics and biomarkers associated with POAF in univariate analysis (*p*-value < 0.05) were included in multiple logistic regression analysis of POAF occurrence.

Term	OR (95% CI)	*p*-Value
Age, per 10 years	2.34 (1.68, 3.27)	**<0.001**
ACE Inhibitor	0.38 (0.17, 0.88)	**0.024**
Angiotensin Receptor Blocker	1.58 (0.66, 3.79)	0.301
Valve Surgery	2.48 (1.18, 5.17)	**0.016**
Percent Δ Nitrite (mean), T = 0 to T = 24 h	0.39 (0.15, 0.99)	**0.047**
BNP at T = 0 h, per 100 pg/mL	1.01 (0.94, 1.09)	0.792

**Table 5:** POAF Prediction Model. Clinical characteristics and biomarkers associated with POAF in univariate analysis with *p*-value < 0.25 were considered as potential features in the POAF prediction model. Feature selection was performed with stepwise logistic regression using the minimum Bayesian information criterion. Fivefold cross-validation was then performed to construct the prediction model.

Term	Estimate	Odd’s Ratio	95% CI	*p*-Value
Intercept	−7.45			
Age, per 10 years	0.89	2.43	(1.71, 3.46)	**<0.001**
ACE Inhibitor	−1.16	0.31	(0.14, 0.72)	**0.006**
Valve Surgery	1.02	2.78	(1.26, 6.13)	**0.011**
IL-6, at T = 48 h	0.01	1.01	(1, 1.01)	**0.022**
Percent Δ Nitrite (mean), T = 0 to T = 24 h	−0.99	0.37	(0.14, 1.02)	0.054

**Table 6:** Biomarkers by AKI Occurrence. Median values are reported for plasma nitrite, percent change in plasma nitrite, IL-6, and BNP levels by POAF occurrence for each listed time point. *p*-values have been calculated using the Mann-Whitney-U test for all biomarkers except percent change in plasma nitrite. Percent change in plasma nitrite from baseline to postoperative day 1 was normally distributed (Kolmogorov-Smirnov normality test *p* > 0.05) thus the *p*-value was calculated using Student’s *t*-test assuming unequal variance.

	No AKI				Stage 1 + AKI				
Serum Biomarker	Sample Size	25th Percentile	Median	75th Percentile	Sample Size	25th Percentile	Median	75th Percentile	*p*-Value
Nitrite, at T = 0 h	154	3.1	4.6	8.8	45	3.3	4.8	9.4	0.859
Nitrite, at T = 24 h	157	2.0	3.0	5.2	47	1.9	2.6	5.9	0.600
Nitrite, at T = 48 h	138	2.0	3.1	5.9	44	1.9	2.8	5.0	0.335
Nitrite, at T = 72 h	77	2.0	2.7	5.1	29	1.9	2.7	4.9	0.935
% Δ Nitrite, T = 0 to T = 24 h	154	−59.2%	−34.6%	−10.7%	45	−57.7%	−41.7%	−25.3%	0.351
IL-6, at T = 0 h	156	1.7	2.5	4.9	43	2.9	4.4	7.1	**<0.001**
IL-6, at T = 24 h	154	36.0	70.7	115.8	44	32.6	98.4	190.6	0.106
IL-6, at T = 48 h	135	37.8	68.2	104.8	44	23.0	77.3	142.2	0.700
IL-6, at T = 72 h	79	25.2	35.4	47.2	29	34.5	61.8	82.2	**0.002**
BNP, at T = 0 h	156	27.8	63.5	145.0	45	69.0	156.0	345.0	**<0.001**

**Table 7:** Multiple Logistic Regression of Clinical and Biomarker AKI Predictors. Clinical characteristics and biomarkers associated with stage 1 or greater AKI in univariate analysis (*p*-value < 0.05) were included in multiple logistic regression analysis of AKI occurrence.

Term	OR (95% CI)	*p*-Value
Age, per 10 years	1.17 (0.83, 1.65)	0.379
Preoperative Creatinine	1.17 (0.78, 1.75)	0.442
Diabetes Mellitus	2.33 (1.01, 5.34)	**0.046**
Beta Blocker	1.67 (0.75, 3.69)	0.206
Statin	1.73 (0.65, 4.63)	0.275
IL-6, at T = 0 h	1.06 (1, 1.12)	0.069
BNP, at T = 0 h, per 100 pg/mL	1.03 (0.96, 1.12)	0.397

**Table 8:** AKI Prediction Model. Clinical characteristics and biomarkers associated with stage 1 or greater AKI in univariate analysis with *p*-value < 0.25 were considered potential features in the AKI prediction model. Feature selection was performed with stepwise logistic regression using the minimum Akaike information criterion. Fivefold cross-validation was then performed to construct the prediction model.

Term	Estimate	Odd’s Ratio	95% CI	*p*-Value
Intercept	−3.23			
Diabetes Mellitus	1.55	4.73	(2.15, 10.42)	**<0.001**
IL-6, at T = 0 h	0.08	1.08	(1.02, 1.15)	**0.010**
Cardiopulmonary Bypass Time, per 10 min	0.07	1.07	(1.02, 1.13)	**0.013**

## Data Availability

The original contributions presented in the study are included in the article, further inquiries can be directed to the corresponding authors.

## References

[1] Ball L., Costantino F., Pelosi P. (2016). Postoperative complications of patients undergoing cardiac surgery. Curr. Opin. Crit. Care.

[2] Pahwa S., Bernabei A., Schaff H., Stulak J., Greason K., Pochettino A., Daly R., Dearani J., Bagameri G., King K. (2021). Impact of postoperative complications after cardiac surgery on long-term survival. J. Card. Surg..

[3] Dobrev D., Aguilar M., Heijman J., Guichard J.-B., Nattel S. (2019). Postoperative atrial fibrillation: Mechanisms, manifestations and management. Nat. Rev. Cardiol..

[4] Wang Y., Bellomo R. (2017). Cardiac surgery-associated acute kidney injury: Risk factors, pathophysiology and treatment. Nat. Rev. Nephrol..

[5] Hobson C.M., Ozrazgat-Baslanti T., Kuxhausen A.B., Thottakkara P.M.E., Efron P.A., Moore F.A., Moldawer L.L., Segal M.S., Bihorac A. (2015). Cost and Mortality Associated With Postoperative Acute Kidney Injury. Ann. Surg..

[6] O’Neal J.B., Shaw A.D., Billings F.T. (2016). Acute kidney injury following cardiac surgery: Current understanding and future directions. Crit. Care.

[7] Khwaja A. (2012). KDIGO clinical practice guidelines for acute kidney injury. Nephron Clin. Pr..

[8] Jawa R.S., Anillo S., Huntoon K., Baumann H., Kulaylat M. (2011). Interleukin-6 in surgery, trauma, and critical care part II: Clinical implications. J. Intensive Care Med..

[9] Prondzinsky R., Knüpfer A., Loppnow H., Redling F., Lehmann D.W., Stabenow I., Witthaut R., Unverzagt S., Radke J., Zerkowski H.-R. (2005). Surgical trauma affects the proinflammatory status after cardiac surgery to a higher degree than cardiopulmonary bypass. J. Thorac. Cardiovasc. Surg..

[10] Zhang Y., Murugesan P., Huang K., Cai H. (2020). NADPH oxidases and oxidase crosstalk in cardiovascular diseases: Novel therapeutic targets. Nat. Rev. Cardiol..

[11] Siu K.L., Lotz C., Ping P., Cai H. (2015). Netrin-1 abrogates ischemia/reperfusion-induced cardiac mitochondrial dysfunction via nitric oxide-dependent attenuation of NOX4 activation and recoupling of NOS. J. Mol. Cell. Cardiol..

[12] Zhang J., Youn J.Y., Kim A.Y., Ramirez R.J., Gao L., Ngo D., Chen P., Scovotti J., Mahajan A., Cai H. (2012). NOX4-Dependent Hydrogen Peroxide Overproduction in Human Atrial Fibrillation and HL-1 Atrial Cells: Relationship to Hypertension. Front. Physiol..

[13] Zhang Y., Shimizu H., Siu K.L., Mahajan A., Chen J.-N., Cai H. (2014). NADPH oxidase 4 induces cardiac arrhythmic phenotype in zebrafish. J. Biol. Chem..

[14] Bryan N.S., Grisham M.B. (2007). Methods to detect nitric oxide and its metabolites in biological samples. Free Radic. Biol. Med..

[15] Mathew J.P., Fontes M.L., Tudor I.C., Ramsay J., Duke P., Mazer C.D., Barash P.G., Hsu P.H., Mangano D.T. (2004). A multicenter risk index for atrial fibrillation after cardiac surgery. JAMA.

[16] Shi N., Liu K., Fan Y., Yang L., Zhang S., Li X., Wu H., Li M., Mao H., Xu X. (2020). The Association Between Obesity and Risk of Acute Kidney Injury After Cardiac Surgery. Front. Endocrinol..

[17] Hofer I.S., Gabel E., Pfeffer M., Mahbouba M., Mahajan A. (2016). A Systematic Approach to Creation of a Perioperative Data Warehouse. Obstet. Anesth. Dig..

[18] Bursac Z., Gauss C.H., Williams D.K., Hosmer D.W. (2008). Purposeful selection of variables in logistic regression. Source Code Biol. Med..

[19] Kuhn M. (2008). Building Predictive Models in R Using the caret Package. J. Stat. Softw..

[20] Robin X., Turck N., Hainard A., Tiberti N., Lisacek F., Sanchez J.-C., Müller M. (2011). pROC: An open-source package for R and S+ to analyze and compare ROC curves. BMC Bioinform..

[21] Wickham H. (2016). ggplot2: Elegant Graphics for Data Analysis.

[22] Jain M.K., Ridker P.M. (2005). Anti-inflammatory effects of statins: Clinical evidence and basic mechanisms. Nat. Rev. Drug Discov..

[23] Rikitake Y., Liao J.K. (2005). Rho GTPases, statins, and nitric oxide. Circ. Res..

[24] Maesen B., Nijs J., Maessen J., Allessie M., Schotten U. (2012). Post-operative atrial fibrillation: A maze of mechanisms. Europace.

[25] Rungatscher A., Tessari M., Stranieri C., Solani E., Linardi D., Milani E., Montresor A., Merigo F., Salvetti B., Menon T. (2015). Oxygenator Is the Main Responsible for Leukocyte Activation in Experimental Model of Extracorporeal Circulation: A Cautionary Tale. Mediat. Inflamm..

[26] Youn J.-Y., Zhang J., Zhang Y., Chen H., Liu D., Ping P., Weiss J.N., Cai H. (2013). Oxidative stress in atrial fibrillation: An emerging role of NADPH oxidase. J. Mol. Cell. Cardiol..

[27] Jayaram R., Goodfellow N., Zhang M.H., Reilly S., Crabtree M., De Silva R., Sayeed R., Casadei B. (2015). Molecular mechanisms of myocardial nitroso-redox imbalance during on-pump cardiac surgery. Lancet.

[28] Kaireviciute D., Blann A.D., Balakrishnan B., Lane D.A., Patel J.V., Uzdavinys G., Norkunas G., Kalinauskas G., Sirvydis V., Aidietis A. (2010). Characterisation and validity of inflammatory biomarkers in the prediction of post-operative atrial fibrillation in coronary artery disease patients. Thromb. Haemost..

[29] Sandler N., Kaczmarek E., Itagaki K., Zheng Y., Otterbein L., Khabbaz K., Liu D., Senthilnathan V., Gruen R.L., Hauser C.J. (2018). Mitochondrial DAMPs Are Released During Cardiopulmonary Bypass Surgery and Are Associated With Postoperative Atrial Fibrillation. Heart Lung Circ..

[30] Zhang W.R., Garg A.X., Coca S.G., Devereaux P.J., Eikelboom J., Kavsak P., McArthur E., Thiessen-Philbrook H., Shortt C., Shlipak M. (2015). Plasma IL-6 and IL-10 Concentrations Predict AKI and Long-Term Mortality in Adults after Cardiac Surgery. J. Am. Soc. Nephrol..

[31] Kumar N., Garg N. (2019). Acute kidney injury after aortic valve replacement in a nationally representative cohort in the USA. Nephrol. Dial. Transplant..

